# Kikuchi disease complicated with aseptic meningitis following COVID-19 Vaccination: a case report

**DOI:** 10.1186/s13256-024-04541-z

**Published:** 2024-06-06

**Authors:** Manana Dewage Sankani Vishvara Kularathna, Arjuna Medagama, Ruwanthi Bandara, Duminda Yasarathna, Madugeta Kumarage Ishara Dilani, Thushani Anuththara

**Affiliations:** Teaching Hospital Peradeniya, Peradeniya, Kandy, Sri Lanka

**Keywords:** Kikuchi Fujimoto disease, Aseptic meningitis, Lymphocytic pleocytosis

## Abstract

**Background:**

Kikuchi Fujimoto disease is a rare self-limiting disorder mainly affecting young Asian females. The typical presentation is unexplained fever with associated cervical lymphadenopathy. It can mimic many sinister diseases such as lymphoma, tuberculosis, and systemic lupus erythematosus. Aseptic meningitis due to Kikuchi disease is extremely rare, and majority were reported from Japan. There have been no published cases of aseptic meningitis due to Kikuchi disease in Sri Lanka.

**Case presentation:**

A 29 years old Sri Lankan female presented with a prolonged fever for three weeks with an associated headache for five days duration. She developed painful cervical lymphadenopathy during the hospital stay. She has been previously well and had been vaccinated against COVID-19 six weeks before. Her lumbar puncture showed lymphocytic pleocytosis with marginally elevated protein levels and reduced ratio of serum to CSF sugar. Lymph node biopsy was consistent with necrotizing lymphadenitis. She was subsequently diagnosed with Kikuchi disease complicated with aseptic meningitis. She responded to corticosteroids well and had an uneventful recovery.

**Conclusion:**

Kikuchi disease is a rare self-limiting disorder that can be complicated with aseptic meningitis on infrequent occasions. Other conditions such as tuberculosis, lymphoma, systemic lupus erythematosus, and adult-onset Still’s disease should be considered as differential diagnoses. Knowledge of Kikuchi disease and its complications will prevent unnecessary investigations which delay the early diagnosis and treatment.

## Introduction

Kikuchi Fujimoto disease is a benign self-limiting disorder commonly affecting young Asian females [1]. It was first described in Japan in 1972 [2]. The most common clinical presentation is unexplained fever and lymphadenopathy, mainly affecting the cervical lymph node group [3]. Skin rashes, gastrointestinal symptoms, and splenomegaly are the main extra nodal manifestations. Rarely, neurological complications are reported with Kikuchi disease [1]. Aseptic meningitis, mono neuritis multiplex, and cerebellar ataxia are among them. According to a meta-analysis of 244 cases of Kikuchi disease, published in 181 case reports, the incidence of neurological manifestations is 11%. Aseptic meningitis had the highest incidence of 2.8–9.8% of all Kikuchi cases [4].

Due to the variety of systemic symptoms, lymphoma, systemic infections such as tuberculosis, inflammatory conditions as Still disease, and systemic lupus erythematosus are considered as differential diagnoses for Kikuchi disease [6]. Most of the Kikuchi disease patients recover spontaneously with symptomatic management and the usage of non-steroidal anti-inflammatory medications. Only a few patients need immunosuppression with steroids [7]. Aseptic meningitis in Kikuchi disease can be mistaken for tuberculous meningitis, primarily when corticosteroids are used as adjuvant therapy [5]. Therefore, the treating physician needs to be aware of this condition to early diagnosis and limit unnecessary investigations and treatments.

Most of the aseptic meningitis associated with Kikuchi disease were reported in Japan [5], with only very few reports of aseptic meningitis outside of Japan. To our knowledge, this is the first case of Kikuchi disease-associated aseptic meningitis to be reported from Sri Lanka.

## Case presentation

We present a 29-year-old Sri Lankan female doctor who presented to Teaching Hospital Peradeniya, with a history of on and off fever for 23 days. It was a low-grade fever that occurred several times per day without chills or rigors and was associated with arthralgia and myalgia. She was well in between fever spikes, and she did not complain of loss of appetite or loss of weight. Fever was associated with headache for five days duration. It was gradually worsening in severity with minimal response to paracetamol. There was associated phonophobia, but no photophobia, nausea, or vomiting. She had a background history of migraine, and the last attack was in 2018.

She was diagnosed with asymptomatic COVID-19 in October 2020 and was subsequently vaccinated with Oxford AstraZeneca (AZD-1222) vaccine. Two weeks after the 2nd dose, she got fever with left axillary pain, and was noted to have left side axillary lymphadenopathy on self examination. The pain and the nodes regressed within two days. She was clinically well for the following six weeks, until presenting with this episode of acute febrile illness. There was no history of sexual risk exposure, prior blood transfusions, or needle stick injuries. She had no features of connective tissue disorders, history of tuberculosis or unacceptable risk exposure.

On examination, she was a small built female with a body mass index of 20 kg/m^2^. She was febrile (39.3 ℃), ill and was in pain. She was not pale or icteric, and there were no skin rashes. She had no neck stiffness or focal neurological signs. The pupils were equally reactive to light. Her lung fields were clear, and there was no hepatosplenomegaly or lymphadenopathy on presentation. Her hemodynamic parameters were stable, with a pulse rate of 88 beats per second and a blood pressure of 110/70 mmHg.

Her investigation results were as follows,

**Table:** 

	Investigation	Results	Normal range
Hematology	Hemoglobin	10.1 g/dL	11–16 g/dL
	White blood cell count	5.31*10^3^/µL	4–10*10^3^/µL
	Neutrophils	2.09*10^3^/µL	2–7*10^3^/µL
	Lymphocytes	2.34*10^3^/µL	0.8–4*10^3^/µL
	Platelet count	140*10^3^/µL	150–450*10^3^/µL
Lumbar puncture	Total cell count	48/mm^3^	< 5/mm^3^
	Polymorphs	25%	
	Lymphocytes	75%	100%
	Total proteins	50.6 mg/dL	15–45 mg/dL
	Sugar	2.5 mmol/L	2.8–4.2 mmol/L (> 50% of plasma level)
	Adenosine deaminase	Negative	
	Tuberculosis culture	Negative	
Biochemistry	Random blood sugar	6.4 mmol/L	< 7.8 mmol/L
	Serum creatinine	102 µmol/L	59–104 µmol/L
	Serum alanine transaminase	62 U/L	0–41 U/L
	Serum aspartate transaminase	32 U/L	0–38 U/L
	Lactate dehydrogenase	620 U/L	140–280 U/L
	Total bilirubin	0.7 mg/dL	0–1.4 mg/dL
	Albumin	30 g/L	34–54 g/L
	Globulin	25 g/L	20–39 g/L
	Ferritin	1704 ng/mL	10–291 ng/mL
	C reactive protein	49.7 mg/L	0–10 mg/L
	Erythrocyte sedimentation rate	42 mm/hour	0–15 mm/hour
	Creatine phosphokinase	36 U/L	24–195 U/L
Serology	Antinuclear antibody 1	> 1:80	
	Antinuclear antibody 2	Negative	
	Anti -ds DNA	Negative	
	Complement component 3	133.9 mg/dL	90–180 mg/dL
	Complement component 4	51.3 mg/dL	10–40 mg/dL
	Human Immunodeficiency virus 1 and 2 antibodies	Negative	
	Anti-EBV antibody IgM	Negative	
	VDRL	Non-reactive	
Microbiology	Blood culture	Negative	
	Urine culture	Negative	
	CSF culture	Negative	
	Sputum for Acid Fast Bacilli	Negative	
	Widal slide agglutination test (SAT)	Negative	
Other	2D echo-	No evidence of endocarditis	
	Blood picture	Viral infection	
	Urine full report	Normal	
	Mantoux	Negative	

As her CSF was compatible with partially treated bacterial meningitis, she was started on intravenous ceftriaxone 2 g bd. Initially, she showed mild clinical improvement but developed spiking fever with painful axillary (mainly left side) and cervical lymphadenopathy. She did not have other organomegaly at that time. Ultrasound scan of the neck and axilla showed enlarged lymph nodes with relatively preserved architecture with thick cortex in bilateral level II and level I B with largest measuring 1.5 cm * 1.1 cm. Her abdomen was ultrasonically normal. Contrast-enhanced CT showed prominent, nonspecific, axillary, para-aortic, and mesenteric lymph nodes with enlarged, oval-shaped, peripherally enhancing cervical lymph nodes at level II B on the right side with suspicion of early suppuration or necrotizing lymphadenitis. Axillary lymph node biopsy showed nodal tissue with preserved architecture. Further, necrotizing regions with histiocytes and numerous karyorrhexis was noted in para-cortical distribution. Hyperplastic follicles were noted. Granulomas were not present. Evidence of Lymphoma was not present. The appearance of necrotizing lymphadenitis, more in favor of post-viral Kikuchi's disease was concluded and systemic lupus erythematosus, drug hypersensitivity, and other vasculitides had been suggested as differential diagnoses. Her bone marrow biopsy showed reactive marrow and Mantoux test, and the sputum for acid-fast bacilli was negative. The vasculitic screening was negative.

**Fig. 1:**
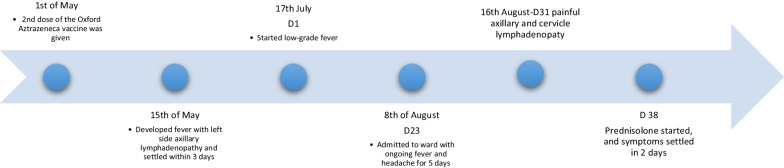
Timeline

On day 38 of the illness, she was started on 45 mg of prednisolone daily. Two days after the commencement of steroids, she showed a good clinical response with reduction of the size of lymph nodes, settling of headache and fever. She was discharged with a diagnosis of Kikuchi disease complicated with aseptic meningitis, which was supported by lymphocytic pleocytosis with a negative CSF culture and necrotizing lymphadenitis in lymph node biopsy (Fig. 1).

## Discussion

This is a case of a young female who presents with aseptic meningitis secondary to Kikuchi disease. It is a less prevalent disorder and mainly occurs in Japan and other surrounding Asian countries [2]. It is a self-limiting disorder that occurs sub-acutely and commonly presents with fever with cervical lymphadenopathy [6]. Lymphoma, tuberculosis, systemic lupus erythematosus, and adult-onset Still’s disease are the main differential diagnoses for Kikuchi disease [3]. While the unilateral involvement of the posterior cervical lymph nodes is the most typical presentation of the disease, a few other uncommon presentations cause diagnostic difficulty [8].

The pathogenesis of Kikuchi disease is not well understood. It has been identified that the onset of the disease is viral or autoimmune in origin, where the defect in T cell immunity plays a significant part in pathogenesis [9]. Epstein Bar Virus, Cytomegalovirus, Parvo B 19 virus, Human immune Deficiency Virus, Brucellosis, Bartonella, and Toxoplasmosis are some agents identified as triggering the onset of Kikuchi disease [6].

Very few case reports have reported Kikuchi disease following vaccination. Watanambe et al. described Kikuchi disease in a ten-year-old Japanese girl following simultaneous administration of Japanese encephalitis vaccine and the Human papilloma vaccine [10]. Few case reports have been reported on axillary, supra-clavicular, and cervical lymphadenopathy following COVID 19 vaccination (Pfizer, AstraZeneca, and Moderna) [11–13]. Furthermore, Al Soub et al. described the development of Kikuchi disease 10 days following COVID-19 vaccination (BNT 162b2) first dose [14]. Though our patient did not have any reaction to the first dose of the Oxford AstraZeneca (AZD-1222) vaccination, she developed axillary lymphadenopathy two weeks following the second dose, which persisted for around three days and settled spontaneously. Her primary illness started almost six weeks after the Oxford AstraZeneca (AZD-1222) vaccination. Her ANA, Ds DNA, C3, C4 levels, HIV 1 and 2 antibodies, EBV antibodies were negative. Therefore we could not identify any of the usual triggers for the onset of Kikuchi disease. Hence we believe that vaccination could have contributed as a trigger for the development of the illness.

Neurological manifestations secondary to Kikuchi disease are rare and aseptic meningitis is the commonest neurological manifestation out of them [4]. Meningeal inflammation without the features of bacterial overgrowth in the culture is considered as aseptic meningitis [15]. Approximately 70% of the cases of aseptic meningitis are due to viral agents, commonly enteroviruses. Furthermore, uncultivatable bacterias such as *Mycobacterium tuberculosi*s, *Treponema pallidum*, fungal, protozoa, malignancies, sarcoidosis, autoimmune disease, and drugs are other causes of aseptic meningitis [16]. Only 2.2–9.8% of patients with Kikuchi disease cases are associated with aseptic meningitis [17], and most of them were reported in Japan [18]. In many cases, it has been demonstrated that the lymphadenopathy will occur simultaneously or after the onset of meningitis [17]. In our case, she initially presented with prolonged fever and subsequently developed features of meningitis followed by lymphadenopathy. There have been no published cases of Kikuchi disease cases complicated with aseptic meningitis in Sri Lanka.

In Kikuchi’s disease, there are no notable laboratory results. It is usual to see leucopenia and anaemia with elevated inflammatory markers. The majority of patients of Kikuchi disease also have elevated serum LDH levels, which may reflect liver involvement. This can make the diagnosis process more difficult because it is also frequently seen in lymphomas, which provide a diagnostic challenge. Elevated levels of ferritin is uncommon in Kikuchi disease. The high ferritin level (1704 ng/mL) noted in our patient could be due to disease it self, co-excisting adult onset Stills disease (AOSD) or Kikuchi disease associated with hemophagocytic lymphohistiocytosis (HLH). However, further investigations were not supportive of either AOSD or HLH [6].

According to Sekiguchi et al. meningitis secondary to Kikuchi disease was associated with a low cerebrospinal fluid/serum sugar ratio [17]. This is important in differentiating aseptic meningitis from viral meningitis. Our patient had a reduced CSF/serum sugar ratio with mildly elevated proteins and CSF lymphocytic pleocytosis, which led to the initial assumption of partially treated bacterial meningitis. With the background of prolonged fever and multiple lymphadenopathy, tuberculosis was also strongly suspected. However, CSF Adenosine deaminase and CSF gene Xpert in our patient were negative.

Three histological patterns of lymph node biopsies have been described by Tseng Tong Kuo et al. in a clinical pathology study using 79 patients with Kikuchi disease [19]. Proliferative, necrotizing and xanthomatous are the described types. It had been suggested that these differences could be due to the differences in etiological factors or the different stages of the disease. The typical histological pattern in Kikuchi disease is necrosis in cortical and paracortical areas in an enlarged lymph node with cellular debris with a proliferation of histiocytes and immunoblasts surrounding the necrotic areas with the absence of plasmacytes or granulocytes [20]. Lymphoma is a common misdiagnosis and the absence of plasma cells and granulocytes aid in the differentiation of Kikuchi disease from lymphoma and lymphadenopathy secondary to bacterial or viral infection [20]. The histology report of our patient revealed necrotizing regions with histiocytes and degenerative nuclear fragments in paracortical distribution without granulomas which favors possible post viral Kikuchi disease.

In many instances, Kikuchi disease is a self-limiting disorder that regresses spontaneously in 1–4 months with symptomatic management with antipyretics and analgesics. Unresponsive cases need to be treated with systemic corticosteroids to aid in early recovery [21]. Hydroxychloroquine and intravenous immunoglobulins are some of the specific therapeutic options which have been used with successful outcomes [22–24]. Our patient was not responding to supportive therapy and was started on oral corticosteroids. She showed a dramatic response with the settling of the fever and headache and receding of lymph nodes within two days of commencement of steroids. It was of utmost importance to exclude tuberculosis before starting steroids in this patient as it may aggravate tuberculosis due to immunosuppression with steroids. Conversely, tuberculous meningitis treatment regime includes steroids, and it can reduce the symptoms of Kikuchi disease with a false interpretation of response to anti-tuberculous therapy.

Around 2% of mortality is reported due to Kikuchi disease, and those were mainly due to pulmonary hemorrhages, hemophagocytic syndrome, disseminated intravascular coagulation, and heart failure [23]. Kikuchi disease can recur in 3–4% of cases, and recurrence aseptic meningitis due to Kikuchi disease is also reported in a limited number of cases [4]. Long-term follow-up of patients with Kikuchi disease is advocated as a significant number of patients become ultimately diagnosed as systemic lupus erythematosis [25].

## Conclusion

Kikuchi disease is a rare self-limiting disorder that can mimic many sinister disorders such as lymphomas, tuberculosis, and systemic lupus erythematosus. Definitive diagnosis of the disease will depend on the histological appearance of the lymph node. Clinical suspicion will guide an early diagnosis to avoid unnecessary invasive and non-invasive investigations. Aseptic meningitis is a rare neurological manifestation of Kikuchi disease, and excluding tuberculous meningitis prior to starting treatment with steroids is mandatory. Apart from the recognized triggers on the development of Kikuchi disease, the probability of post-vaccination immune changes triggering the disease should be further evaluated using further observational studies. The patients with Kikuchi disease should be followed up in the long run due to the increased incidence of developing systemic lupus erythematosus.

## Data Availability

Data sharing is not applicable to this article as no datasets were generated or analyzed during the current study.
